# New occurrences, mean infestation intensity and prevalence of parasitic isopods (Isopoda, Cymothoida, Bopyridae) associated with *Macrobrachium amazonicum* (Decapoda, Palaemonidae) from the mouth of the Amazon River

**DOI:** 10.1590/S1984-29612024037

**Published:** 2024-07-15

**Authors:** Sting Silva Duarte, Jô de Farias Lima, Lucio André Viana

**Affiliations:** 1 Programa de Pós-graduação em Biodiversidade Tropical, Universidade Federal do Amapá – UNIFAP, Macapá, AP, Brasil; 2 Embrapa Amapá, Macapá, AP, Brasil; 3 Laboratório de Estudos Morfofisiológicos e Parasitários, Departamento de Ciências Biológicas e da Saúde, Universidade Federal do Amapá – UNIFAP, Macapá, AP, Brasil

**Keywords:** Parasitism, Amazon-prawn, ectoparasites, Parasitismo, Camarão-da-amazônia, ectoparasitos

## Abstract

The Amazon prawn or *Macrobrachium amazonicum* (Heller, 1862) is widely distributed in South America, occurring in the Orinoco and Amazon rivers, and forms an important source of income for riverside families. This prawn hosts crustacean ectoparasites of the genus *Probopyrus* (Giard & Bonnier, 1888) (Bopyridae) that infest its gill cavity. The aim of the present study was to report new occurrences of *Probopyrus* in Amazon prawns caught in the Amazon River. *Macrobrachium amazonicum* prawns were collected between May 2017 and April 2018, and again from July 2021 to May 2022 in the regions of Ilha de Santana and Rio Mazagão, state of Amapá, Brazil. Among the 5,179 prawn specimens caught, 133 were parasitized by the ectoparasites *Probopyrus pandalicola* (Packard, 1879), *Probopyrus bithynis* (Richardson, 1904), *Probopyrus floridensis* (Richardson, 1904) and *Probopyrus palaemoni* (Lemos de Castro & Brasil Lima, 1974). These occurrences of *P. floridensis* and *P. palaemoni* in *M. amazonicum* were the first records of this on the northern coast of Brazil. These four ectoparasites are not limited to specific host species or genera, as observed in this study, which reports four species of *Probopyrus* infesting *M. amazonicum*.

## Resumo

O camarão-da-amazônia ou *Macrobrachium amazonicum* (Heller, 1862) é amplamente distribuído na América do Sul, ocorrendo nos rios Orinoco e Amazonas, importante fonte renda de familias riberinhas. Esse camarão é hospedeiro de ectoparasitas crustáceos do gênero *Probopyrus* (Giard & Bonnier, 1888) (Bopyridae) que infestam a cavidade branquial da espécie. O presente estudo tem o objetivo de relatar novas ocorrências de ectopasitas do gênero *Probopyrus* no camarão-da-amazônia, capturados no rio Amazonas. *Macrobrachium amazonicum* foram coletados no período de maio de 2017 e abril de 2018, e novamente de julho de 2021 a maio de 2022, nas regiões da Ilha de Santana e Rio Mazagão, estado do Amapá, Brasil. Entre 5.179 exemplares de camarões capturados, 133 foram parasitados sendo quatro pelos ectoparasitas *Probopyrus pandalicola* (Packard, 1879), *Probopyrus bithynis* (Richardson, 1904), *Probopyrus floridensis* (Richardson, 1904) e *Probopyrus palaemoni* (Lemos de Castro & Brasil Lima, 1974). Registrado pela primeira vez a ocorrência no *M. amazonicum* por *P. floridensis* e *P. palaemoni* no litoral norte do Brasil. Esses quatro ectoparasitos não se limitam a espécies ou gêneros hospedeiros específicos, observados nesse estudo que relata quatro espécies de *Probopyru*s infestando *M. amazonicum*.

## Introduction

The genus *Macrobrachium* (Bate, 1868) includes species commonly found in lentic and lotic environments (Coelho & Ramos-Porto, 1984) and is widely exploited in both aquaculture and artisanal fisheries. These activities are an important source of economic development in many regions of Latin America (Maciel & Valenti, 2009; García-Guerrero et al., 2015). *Macrobrachium amazonicum* is a prawn that usually inhabits estuarine and freshwater environments in Latin America. It can be found underneath rocks and among aquatic vegetation (Maciel & Valenti, 2009), and is very commonly exploited by artisanal fisheries in many regions of Latin America, including the Amazon region of Brazil (New et al., 2000).

Ectoparasitic isopods from the family Bopyridae usually infect prawns as their definitive hosts and remain attached to the prawns’ branchial chambers (Beck, 1980; Chaplin-Ebanks & Curran, 2007). Parasitic interactions between isopods of Bopyridae and prawns are common and reasonably well known in the scientific literature, mainly because many parasitic isopods are crustacean-specific and feed on their hemolymph (Walker, 1977; Boxshall et al., 2005). This happens in many species of the genus *Macrobrachium* (Collart, 1990; Román-Contreras, 1996; Masunari et al., 2000; Conner & Bauer, 2010; Vargas-Ceballos et al., 2016). The impacts from these infestations can include castration, feminization in males (specifically consisting of impairment of claw growth and modification of growth patterns) and behavioral and metabolic disturbances (Neves et al., 2000; Lester, 2005; Chaplin-Ebanks & Curran, 2007; Calado et al., 2008; Dumbauld et al., 2011).

Information on the intensity of infection, specificity and even the geographic range of ectoparasitic isopods from the family Bopyridae is scarce for most species, compared with the number of reports on their taxonomy. Only six species of Bopyridea and one of Entoniscidae have been recorded from 10 host species (Román-Contreras, 2008; Román-Contreras & Martínez-Mayén, 2011). The apparently low number of bopyrids in the Brazil region is probably due to limited sampling effort (Shields et al., 2015) or to their omission from studies on the taxonomic and ecological aspects of their hosts (Boyko & Williams, 2009). In this paper, we register mean infection intensity, prevalence and a new locality and host record for ectoparasitic species of the genus *Probopyrus*, associated with *Macrobrachium amazonicum* captured from the mouth of the Amazon river.

## Material and Methods

**Figure 1:**
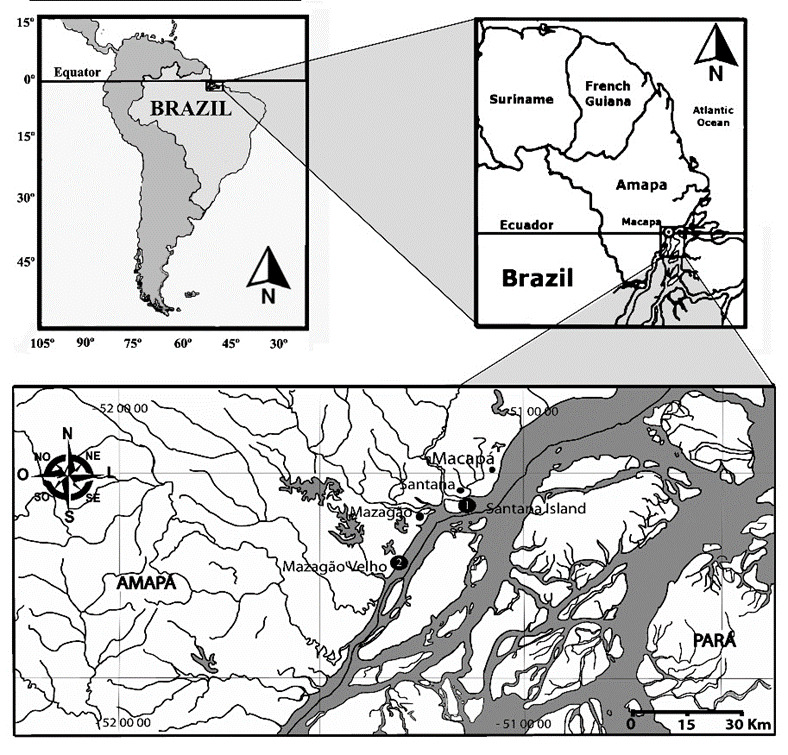
Locations where *Macrobrachium amazonicum* was caught at the mouth and lower reaches of the Amazon River between May 2017 and April 2018, and between July 2021 and May 2022. 1) Santana Island, 2) Mazagão Velho.

We collected, *M. amazonicum* specimens from Santana Island (00º03'40.9'' S and 051º08'46.6'' W) and Mazagão Velho (00º15'39.9'' S and 051º20'42.3' W), both located in the estuary of the Amazon River in the state of Amapá, Brazil (as shown in Figure 1). Specimens of *M. amazonicum* were collected using 40 pit traps between May 2017 and April 2018, and again from July 2021 to May 2022, with 20 traps in each study region. The organisms thus caught were transported alive in containers filled with water to the laboratory and were stored at -20°C until required. Prawns identification was carried out using the taxonomic keys of Holthuis (1952) and Melo et al. (2003).

The sex of prawns was determined by examining the second pair of pleopods. The presence of the male appendix indicates a male and its absence indicates a female (Ismael & New, 2000; Carvalho et al., 2014). A bulge in the exoskeleton at the gill chamber can identify prawns infected with parasites. To determine the prevalence and mean infection intensity of parasitized prawns at each collection site, the total number of hosts examined for the specific parasite species or taxonomic group of infected hosts is divided by the total number of hosts examined recommended Bush et al. (1997). The parasites were identified based on the original descriptions presented by Muñoz (1997), Lemos de Castro & Loyola e Silva (1985), Román-Contreras (2004) and Paul et al. (2010). References are provided for the taxonomic authority of all parasitic taxa, but not for those of hosts.

## Results

Overall, we caught a total of 5,381 specimens of *M. amazonicum*, consisting of 2,975 females, 2,195 males and 211 specimens of unknown sex. The sex ratio of females to males was 1.35 to 1. Among the females sampled, 1,045 carried eggs in their abdomen and 1930 did not. Ovigerous females ranged in carapace length from 11.10 to 29.6 mm, which 52.5% were between the lengths 11.10 and 19.09 mm.

**Figure 2:**
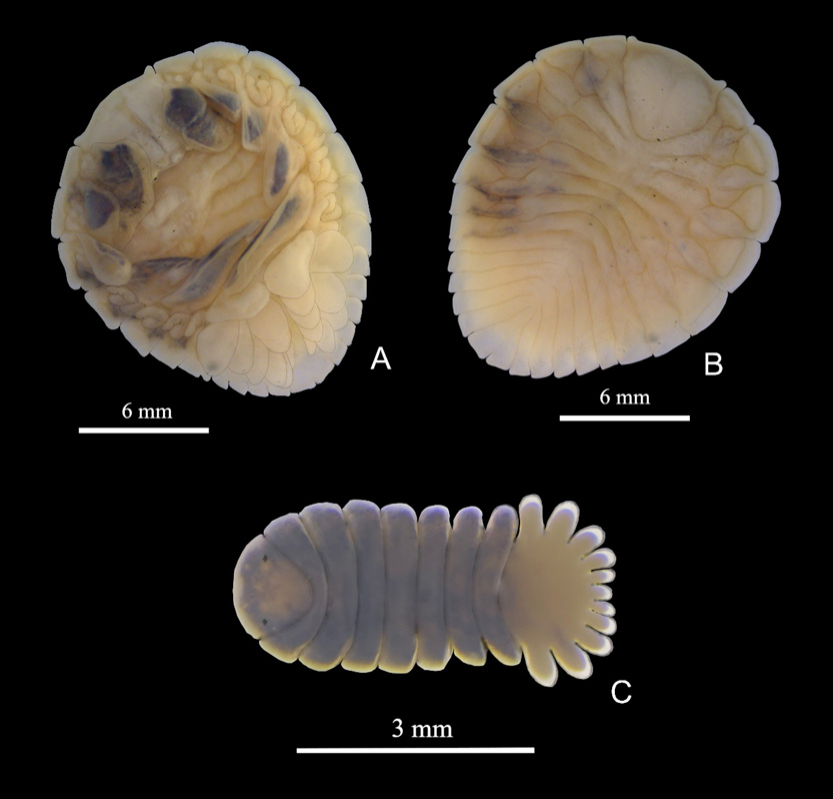
*Probopyrus bithynis.* A – female in ventral view; B – female in dorsal view; C – male in dorsal view.

**Figure 3:**
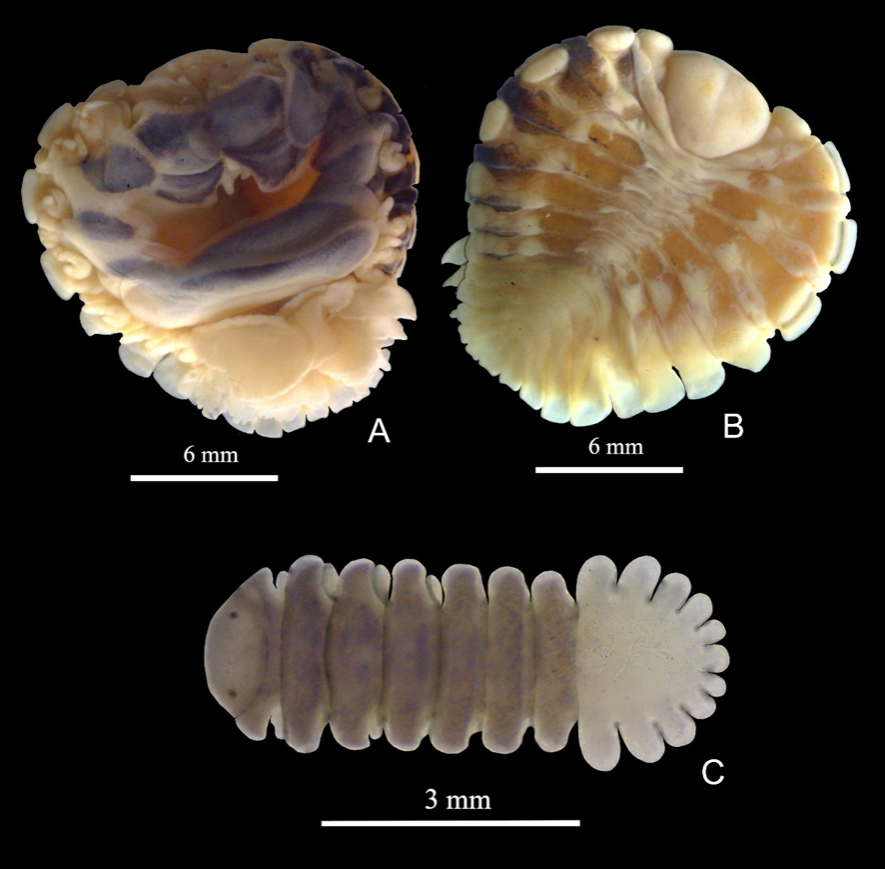
*Probopyrus floridensis.* A – female in ventral view; B – female in dorsal view; C – male in dorsal view.

**Figure 4:**
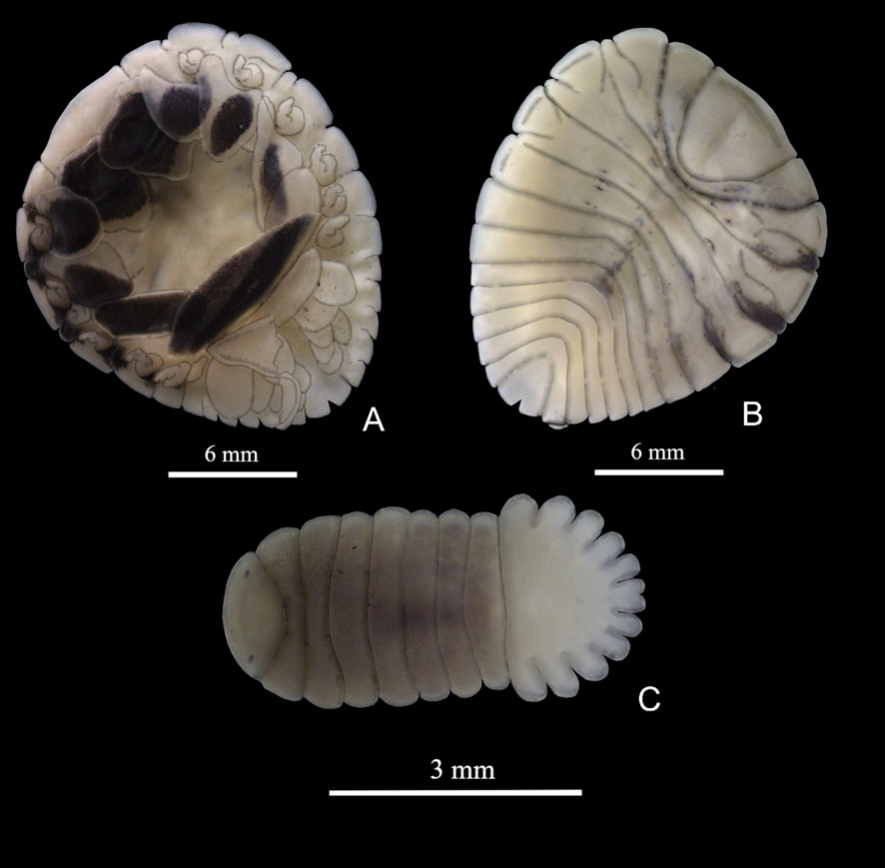
*Probopyrus palaemoni.* A – female in ventral view; B – female in dorsal view; C – male in dorsal view.

**Figure 5:**
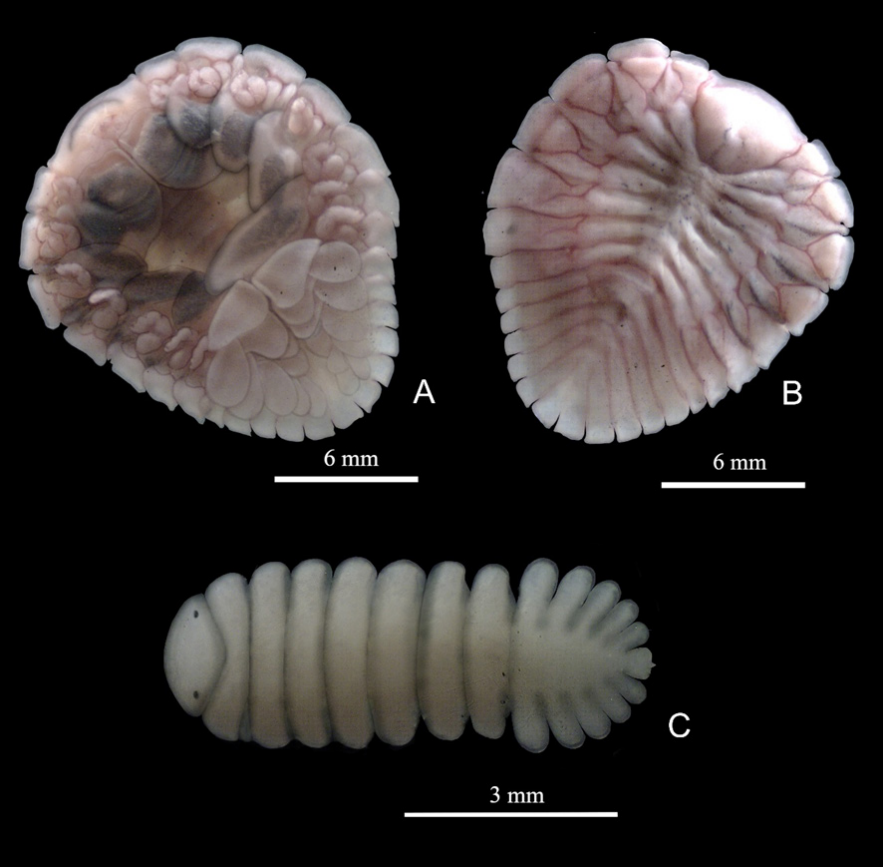
*Probopyrus pandalicola.* A – female in ventral view; B – female in dorsal view; C – male in dorsal view.

Among the total number of prawns sampled, 133 specimens were infested with different developmental stages of bopyrids, of which 20% were *Probopyrus bithynis* Richardson, 1904, 17% were *Probopyrus floridensis* Richardson, 1904, 30% were *Probopyrus palaemoni* Lemos de Castro & Brasil Lima, 1974, and 31% were *Probopyrus pandalicola* (Packard, 1879) (Figures 2, 3, 4 and 5). The prevalence and mean infection intensity were low, of which 0.50 were *Probopyrus bithynis*, 0.42 were *Probopyrus floridensis*, 0.76 were *Probopyrus palaemoni* and 0.78 were *Probopyrus pandalicola*. For the mean infection intensity, of which 1.0 were *Probopyrus bithynis*, 1.0 were *Probopyrus floridensis*, 1.0 were *Probopyrus palaemoni* and 1.0 were *Probopyrus pandalicola*.

The majority of the parasitized prawns were female (75%), and none of the ovigerous females were found to be parasitized. Most of the parasitized prawns had only one gill chamber infected (98.1%), while 1.9% had both chambers infected. The characteristics of the parasites reported in this study were concordant with the original descriptions and others reported in the specialized literature, and their systematic positions are presented below:

Genus *Probopyrus* Giard & Bonnier, 1888. Type species: *Bopyrus ascendens* Semper, 1880, accepted as *Probopyrus ascendens* (Semper, 1880) (through original designation).

### *Probopyrus bithynis* Richardson, 1904

*Probopyrus bithynis* Richardson, 1904: 68–70, figs. 46–51. —Richardson, 1905a: 555, 557–559, figs. 606–611. —Pearse, 1911: 109. —Pearse, 1915: 550. —Chopra, 1923: 508, 510. —Nierstrasz & Brender à Brandis, 1923: 94. —Van Name, 1925: 481–484. —Van Name, 1936: 32, 38, 485–489, figs. 309–311. —Cordero, 1937: 10–11. —Mackin & Hubricht, 1938: 634. —Carvalho, 1942: 129, 131. —Rioja, 1949: 172–173. —Holthuis, 1950: 120. —Pennak, 1953: 430, fig. 269. —Chace et al., 1959: 873, fig. 31.2. —Green, 1961: 143. —Schultz, 1969: 331, fig. 534a. —Kaestner, 1970: 463. —Coelho & Koenig, 1972: 256, table I. —Lemos de Castro & Brasil Lima, 1974: 209, 214–216, figs. 16–26.—Truesdale & Mermilliod, 1977: 217–219, fig. 1, table I. —Bourdon, 1979: 501. —Anderson & Dale, 1981: 156. —Dale & Anderson, 1982: 392–396, 402–404, 406, 407, figs. 1–4, tables 1–2, 4. —Markham, 1985: 26–28. —Markham, 1988: 38 (mention). — Verdi, 1991: 335–339. —Román-Contreras, 1993: 689–690, 694–695. —Brasil-Lima, 1998: 638 (list). —Rocha & Bueno, 2000: 133–134, 137–138. —Román-Contreras & Bourdon, 2001: 920 (table 1), 922 (table 2). —Raman et al., 2005: 29–37, fig. 1. —Saito et al., 2010: 179–180. —Román-Contreras & Martinez-Mayén, 2011: 1150. —Corrêa et al., 2018: 119, fig. 2. —Ribeiro et al., 2019: 2443–2444 (mention). —Aguilar-Perera, 2022: 116 (table). —Pereira et al., 2022: 203, 206–207. — Ribeiro & Horch, 2023: 157-158 (mention).

Hosts: prawn species (infraorder Caridea): *Macrobrachium ohione* (Smith, 1874), *M. olfersii* (Wiegmann, 1836), *M. acanthurus* (Wiegmann, 1836), *M. amazonicum* (Heller, 1862), *M. borellii* (Nobili, 1896), *M. rosenbergii* (De Man, 1879) (Markham, 1985; Verdi, 1991; Raman et al., 2005).

Distribution: Western Atlantic: United States (Louisiana, Missouri, Mississippi), West Indies, Mexico, Nicaragua, Colombia, Guyana, Brazil (states of Amapá, Pará, Rio Grande do Norte, Paraíba), Uruguay, Argentina (Buenos Aires) (Markham, 1985; Verdi, 1991; Brasil-Lima, 1998). Indian Ocean, Bay of Bengal: India (Andhra Pradesh) (Raman et al., 2005).

Diagnosis: In ventral view, the female's body is mainly white on the top, with only three small black patches on one side of the back-lateral parts of the second, third and fourth thoracic segments (Figure 2A). The first pair of incubatory lamellae have dark to black patches, and all other lamellae on one side have these patches except for the second lamella, which may have some. On the other side, the lamellae do not have these patches. Patches of dark are also present on the ventral side of the lateral margins of the second, third and fourth thoracic segments, on the same side as the markings on the dorsal surface. The incubatory lamellae, marked with patches, are attached to these segments. All the lamellae are extensive and encompass the marsupium, leaving only a small opening into the pouch. The legs on both sides are white, without markings, and they show high expansion or carina at the base.

The head has prominent processes in the anterolateral corners. The anterior margin between these processes is straight, while the posterior margin is narrowly rounded. The length of the head is about equal to its breadth, eyes are not visible. The epimera are visible as narrow pieces lateral to the ovarian bosses on all segments. In dorsal view, the abdominal segments are distinct (Figure 2B). The lateral margins of the first five segments are straight. The sixth or terminal segment is narrow and elongated.

In dorsal view, the male has segments that are not widely separated at the side. The male has eyes and brown markings. The body is short and thick, only twice as long as it is wide (as shown in Figure 2C). The abdomen is a little more than one and a half times broader than it is long. The segments of the abdomen are only visible at the side, as they are fused in the middle of the dorsal surface. They gradually decrease in size to the sixth and last segment, which is a narrow piece between the two lobes of the fifth segment that does not extend to the extremity of those lobes.

Remarks: *Probopyrus bithynis* was described based on two groups of parasites that showed clear morphological variations, as observed in illustrations and descriptions reported by Richardson in 1904. Later, an analysis on the type and paratype specimens of *P. bithynis* and *P. pandalicola* (Markham, 1985) suggested that these species are synonyms, based on the morphological similarity. However, this hypothesis was then discarded and *P. bithynis* was re-established as a valid species by Román-Contreras in 1993, in agreement with Dale & Anderson in 1982.

Based on the illustrations and descriptions presented by Richardson in 1904, we propose that the specimens that differed from the type *P. bithynis* are different species. We observed that the absence of anterolateral processes on the head of the female, the presence of patches of black on the lateral margins of all the segments of the thorax on one side of the body, and the presence of a bilobed telson are morphologically similar to what was observed in specimens later designated by Lemos de Castro & Brasil Lima in 1974 as *P. palaemoni*.

Recently, Pereira et al. (2022) conducted a study on *Probopyrus sp*. populations that parasitize *Macrobrachium amazonicum*, which were sampled in coastal (Abaetetuba, Afuá, Augusto Corrêa and Breves-state of Pará, Brazil) areas of the Amazon and continental (Santarém-state of Pará, Brazil). They concluded that the coastal populations analyzed could be assigned to *P. bithynis*, while the inland populations consisted of a different species, probably *P. palaemoni*.

### *Probopyrus floridensis* Richardson, 1904

*Probopyrus floridensis* Richardson, 1904a: 70–71, figs. 52–55. —Richardson, 1905: 555–556, figs. 602–605. —Richardson, 1912: 524. —Chopra, 1923: 508–510. —Nierstrasz & Brender à Brandis, 1925: 7. —Van Name, 1925: 483. —Nierstrasz & Brender à Brandis, 1929: 23–24. —Chopra, 1930: 128. —Carvalho, 1942: 125–133, figs. 1–2, pl. I. —Morris, 1948: 1. —Rioja, 1949: 172–173. —Hutton & Sogandares-Bernal, 1960: 287. —Hutton, 1964: 447. —Moore & McCormick, 1969: R88, fig. 33(2a, b, c). —Schultz, 1969: 331, fig. 533. —Lemos de Castro & Brasil Lima, 1974: 209, 212–214, figs. 1–15. —Dale & Anderson, 1982: 392–395, 397–398, 400, 402–407, figs. 2, 7, tables 1–4. —Markham, 1985: 26–27. —Markham, 1988: 38 (mention). —Román-Contreras, 1993: 689–690, 694–695. —Brasil-Lima, 1998: 638 (list). —Masunari et al., 2000: 1095– 1106, fig. 2. —Rocha & Bueno, 2000: 133–138, figs. 2–4. —Román-Contreras & Bourdon, 2001: 920 (table 1), 922 (table 2). —Saito et al., 2010: 179–180. —Román-Contreras & Martinez-Mayén, 2011: 1149 (mention). —Ribeiro et al., 2019: 2443–2444 (mention). —Pereira et al., 2022: 203, 207 (mention). —Ribeiro & Horch, 2023: 158 (mention).

Hosts: prawn species (infraorder Caridea) *Palaemon paludosus* (Gibbes, 1850), *Macrobrachium potiuna* (Müller, 1880) (Brasil-Lima, 1998; Rocha & Bueno, 2000) and *Macrobrachium amazonicum* in the present study.

Distribution: Western Atlantic: United States (Georgia and Florida) and Brazil (states of Espírito Santo, São Paulo, Paraná and Amapá) (Markham, 1985; Brasil-Lima, 1998; Masunari et al., 2000). This is the first report of this parasite in *M. amazonicum* and a new occurrence in northern Brazil.

Diagnosis: The female's body is light brown and has distinct parts such as the head, abdomen, ovarian bosses and light yellow epimera. There are dark markings all over the thorax and a few black lines on the abdomen. The incubatory lamellae are almost entirely covered with black markings, giving a uniformly dark color. On the ventral side of the thorax, the lateral parts have black markings with yellow areas separating them, and all the legs on this side are yellow (see Figure 3A). The legs on the opposite side are dark.

The head is deeply set in the thorax and has a broad anterior with a straight frontal margin and a narrowly rounded posterior margin. The thorax has distinct segments, and the ovarian bosses are prominent on the anterior portion of the sublateral margin of the first four segments. The epimera are narrow plates located laterally to the ovarian bosses, occupying the lateral margin.

The abdominal segments are distinctly separated on the dorsal side, and the lateral margins are narrowly rounded (see Figure 3B). The terminal segment of the body is long and narrow, rounded posteriorly, and sometimes has a tiny excavation. The pleopoda consist of five pairs of double-branched lamellar appendages. The incubatory lamellae are large and encircle the incubatory pouch, leaving only a tiny opening into the interior. The first pair of plates has a terminal lobe in the distal segment. All legs have a well-rounded expansion or carina around the middle of the base.

The male has well-defined thorax segments that are widely separated at the sides (see Figure 3C). The body is narrow, elongated and nearly three times as long as broad. The abdomen has well-defined segments at the sides but is fused in the middle of the dorsal surface. The terminal segment is well-defined, rounded posteriorly, and extends beyond the lobes of the preceding segment. The lateral margins of all the segments are rounded, and pleopoda are present in the form of pairs of small, rounded processes, with one pair on each segment of the abdomen. The eyes are visible in dorsal view.

Remarks: *Probopyrus floridensis*, like *Probopyrus bithynis*, was initially considered to be a synonym of *P. pandalicola*, by Markham (1985). However, it was later established as a distinct species by Román-Contreras (1993), in agreement with Dale & Anderson (1982). Two studies have been conducted on the population structure and prevalence of this species on the host *Macrobrachium potiuna* in Brazil. One was conducted in Paraná (Masunari et al., 2000), while the other was conducted in São Paulo (Rocha & Bueno, 2000). The study by Masunari et al. (2000) registered the southernmost location known for this species, while Rocha & Bueno (2000) discussed the taxonomic validity of the species. The present study provides the first report of this parasite in *M. amazonicum* from Brazil.

### *Probopyrus palaemoni* Lemos de Castro & Brasil Lima, 1974

*Probopyrus palaemoni* Lemos de Castro & Brasil Lima, 1974: 216–217, figs. 20–26. —Brasil-Lima, 1998: 368 (list). —Rocha & Bueno, 2000: 133 (mention). —Ribeiro et al., 2019: 2444 (mention). —Ribeiro & Horch, 2023: 158 (mention).

Hosts: prawn species (infraorder Caridea): *Palaemon pandaliformis* (Stimpson, 1871), *P. paludosus* (Gibbes, 1850) (Brasil-Lima, 1998) and *Macrobrachium amazonicum* in the present study.

Distribution: Southwestern Atlantic: Brazil (state of Rio de Janeiro) (Brasil-Lima, 1998) and Amapá (present study).

Diagnosis: The female's body is light brown and has distinct parts such as the head, abdomen, ovarian bosses and light yellow epimera. In ventral view, the female's body is light brown with varied dark parts, but more accentuated on the posterior margins of thoracic somites II, III and IV, close to the lateral bars, and more accentuated only on one side (Figure 4A). The head and pleon are depigmented. The head is deeply inserted into the first thoracic segment. The anterior margin is almost straight, forming two short subtriangular processes laterally, and the posterior margin is narrow and rounded.

The lamellae of the marsupium are uniformly pigmented, with the exception of one pair that presents a narrow yellowish interior stripe. The somites of the pereon are distinct, and the first five have prominent ovarian bosses and strongly distinct epimera. There are well-developed marsupial lamellae. The pleon somites are separated, with the ends contiguous to each other and lateral margins slightly curved. There are five pairs of pleopods with both lamellar branches: the first pair is more developed and the others gradually decrease in size.

The telson is slightly wider than long and triangular, narrow at the base and laterally forming two rounded angles. Medianly, it presents a deep V-shaped incision (Figure 4B).

The males’ body is about three times wider than it is long. The pleon is distinctly wider than the pereon. The eyes are visible dorsally. The pereon somites are very distinct and strongly separated laterally. The pleon somites are well-marked laterally, forming rounded lobes, and they end in a bilobed telson posteriorly (Figure 4C).

Remarks: The absence of anterolateral processes on the head of the female, the presence of patches of black on the lateral margins of all the segments of the thorax on one side of the body and the presence of a bilobed telson that were observed in *P. palaemoni* are morphologically similar to what was observed in illustrations and descriptions presented by Richardson in 1904 for a variant of *P. bithynis*. Although in the present study some male specimens present variations in the telson, such that some are more bilobed and others less so, the specimens found in *M. amazonicum* presented the same characteristics as had previously been described for this species.

### *Probopyrus pandalicola* (Packard, 1879)

Abbreviated synonymy (see Markham, 1985 and Ribeiro et al., 2019: the synonymy list below includes only taxonomic resources since 2019).

*Probopyrus pandalicola* Ribeiro et al., 2019: 2440–2444, figs. 2, 3. —De Barros et al., 2021: 273–377, fig. 2. —Aguilar-Perera, 2022: 116 (table). —Pereira et al., 2022: 203, 205–207 (mention), tables 2–3. —Ribeiro & Horch, 2023: 159 (mention).

Hosts: prawn species (infraorder Caridea): *Palaemon vulgaris* Say, 1818 (cited as *Palaemonetes vulgaris*), *P. northropi* (Rankin, 1898), *P. pugio* (Holthuis, 1949), *P. intermedius* (Stimpson, 1860), *P. kadiakensis* (Rathbun, 1902), *P. paludosus* (Gibbes, 1850), *P. pandaliformis* (Stimpson, 1871), *Macrobrachium acanthurus* (Wiegmann, 1836), *M. olfersii* (Wiegmann, 1836), *M. amazonicum* (Heller, 1862), *M. carcinus* (Linnaeus, 1758), *M. surinamicum* Holthuis, 1948, *Cuapetes mericanos* (Kingsley, 1878) (cited as *Periclimenes mericanos*) (Markham, 1985; Ribeiro et al., 2019).

Distribution: Western Atlantic: United States (from New Jersey to Florida, Mississippi and Texas), U.S. Virgin Islands, Mexico, Costa Rica, Cuba, Colombia, Venezuela, Suriname and Brazil (states of Pará, Amapá, Pernambuco, Bahia, and Rio de Janeiro) (Markham, 1985; Ribeiro et al., 2019).

Diagnosis: In ventral view (Figure 5A), the head of the female is wedge-shaped and deeply embedded into the body. Its front edge extends slightly beyond the main body and does not have a frontal plate. The antennae have three parts each, and the last two parts are small in the second antenna. The maxilliped is segmented with short, non-articulated setose palp lateral projections on each side. The middle region is produced into two wide and blunt points, while the inner region is long and slender.

The pereomeres are distinctly separated dorsally and laterally (Figure 5A-B). Dorsolateral bosses on both sides of pereomeres 1-4, coxal plates on short sides of pereomeres 1-4 and on long sides of pereomeres 1-5. Oostegites surrounding but not enclosing brood pouch; oostegite 1 with prominent, slender, subfalcate, posterolateral point and dentate internal ridge. All articles of all pereopods are distinct; the size of pereopods increasing posteriorly; bases proportionately larger and more carinate posteriorly; all dactyls deeply set into propods.

The final segment of the pleon overlaps with the one before it. There are six distinct pleomeres, but they are only slightly separated from each other on the sides (as shown in Figure 5B). The first five pleomeres are nearly the same length and about half the length of the posterior ones. The last pleomere is triangular and truncated posteriorly. The anterolateral margins of the first four pleomeres have reflexed sides. There are five pairs of biramous and foliate pleopods, and the endopodites of the first pair are much more prominent than the others, concealing nearly all other pleopods.

All regions and segments of the male body are distinct dorsally and separated laterally (Figure 5C). The sides of the pereon are subparallel, but the pleon suddenly becomes broader than the pereon. Most of the dorsal surface of the body has dark brown pigment.

Head prominently extended anteriorly, semicircular, with slight concavity in front; posterolateral corners produced into slight points. Large, irregularly shaped eyespots near edges. Antennae each of three articles, smaller distally; antenna 1 with setae on distal margin of each article; setae possibly on antenna 2 but not discernible.

The pereomeres are distinct dorsally and separated by notches laterally; lateral margins of all pereomeres are greatly reflexed ventrally. Abdomen with all somites well developed, fused in the center but separated laterally by deep incisions. Telson with rounded contour and pointed distal end. The pleopods form five pairs of rounded tubercles, one for each pair. Pleopoda are present in pairs of small, rounded processes, one pair on each segment of the abdomen. Eyes are present.

Remarks: According to Ribeiro & Horch (2023), *P. pandalicola* has widespread distribution in Brazil. See Ribeiro et al. (2019) for a discussion on the distribution and taxonomic history of this widespread species. Several studies have cited the southern edge of *P. pandalicola* distribution as the state of São Paulo, Brazil, following Beck (1979). However, it should be noted that Beck (1979) considered *P. floridensis* to be a synonym of *P. pandalicola* and combined their distributions when discussing the species. As far as we have been able to discern through separating the two species in the literature, the southernmost location of *P. pandalicola* is in the state of Rio de Janeiro and the northernmost is in the state of Amapá, Brazil.

## Discussion

There has been much debate surrounding the morphology of *Probopyrus* over recent years, such that the species *Probopyrus floridensis* and *P. bithynis* from the Western Atlantic synonymized with *P. pandalicola* by Markham (1985). However, according to Dale & Anderson (1982), the morphology of larvae belonging to *Probopyrus bithynis*, *P. pandalicola* and *P. floridensis* are distinct. Currently, all three species (*P. bithynis*, *P. pandalicola* and *P. floridensis*) are recognized as valid by the World Register of Marine Species (WoRMS, 2023).

In the present study, we examined 133 specimens infesting *M. amazonicum* from Santana Island and Mazagão River. We were able to distinguish four clear morphological patterns that indicated the species *P. pandalicola*, *P. bithynis*, *P. floridensis* and *P. palaemoni*, based on the original descriptions and illustrations. We record here, for the first time, infestation of *M. amazonicum* by *P. floridensis* and *P. palaemoni* on the north coast of Brazil. According to Pralon et al. (2018), infestations of *Probopyrus* species are not limited to specific host species or genera, and this is supported by the present study that reports four *Probopyrus* species infesting *M. amazonicum*.

In the present study, infestation by *Probopyrus* sp. mainly occurred in females of *M. amazonicum*. This is consistent with other studies conducted on carideans, in which parasite prevalence was found to be higher in females (Chaplin-Ebanks & Curran, 2007; Rasch & Bauer, 2015; Barros et al., 2021) or equal for both sexes (Marin Jarrin & Shanks, 2008). Although Pralon et al. (2018) did not conclude that *Probopyrus* has sex-specific prevalence, due to the low number of parasites found in the specimens that they collected, our data suggest otherwise. We found that species of *Probopyrus* showed higher prevalence in female hosts.

In the present study, *M. amazonicum* showed apparent prevalence of infestations in just one gill chamber, regardless of the species infesting, and this could either be the left or the right one. This is consistent with observations in many other palaemonid species (Collart, 1991; Jiménez & Vargas, 1990; Román-Contreras, 2004; Hassan et al., 2017; Correa et al., 2018; Barros et al., 2021). Although Correa et al. (2018) suggested that it is viable for *Probopyrus* to infest both gill chambers, our results indicate that such occurrences are rare. Female bopyrids puncture the dorsal branchial chamber cuticle of their hosts to obtain hemolymph, which deforms the carapace and infiltrates hemocytes, thus impairing gill function. This can potentially affect the respiratory and osmoregulatory capacity of *M. amazonicum*, as reported by Corrêa et al. (2018). The greater prevalence of infestation in only one of the gill chambers is a survival strategy to keep the host alive.

In the present study, prevalence and mean infection intensity of ectoparasites wares low. May be attributed to variations in environmental conditions such as salinity, which can interfere with the life cycle of intermediate hosts and the varying abundance of intermediate hosts can have a direct impact on the reproductive cycle of the parasites (Román-Contreras, 2004; Hassan et al., 2017; Corrêa et al., 2018; Barros et al., 2021). This suggests that intermediate hosts of the parasites observed in this work can be more commonly found in estuarine areas than in continental ones.

The loss of nutrients through parasite action can disrupt and hinder the production of hormonal compounds that are believed to trigger growth processes and reproduction (Fingerman, 1997; Subramoniam, 2011; Swetha et al., 2011). A study conducted by Sherman & Curran (2015) revealed that prawns infested by *Probopyrus* experienced sexual sterilization. In the present study, none of the *M. amazonicum* females infested by *Probopyrus* were ovigerous. Parasite infestation probably affects the host negatively, regardless of the species. This effect could potentially have a negative influence on recruitment processes, depending on the severity of the infection (Barros et al. 2021).

## Conclusions

This study confirms the occurrence of *P. floridensis*, *P. bithynis*, *P. palaemoni* and *P. pandalicola* in the prawn *M. amazonicum* from the mouth of the Amazon river, Brazil. It showed that the parasite has a preference for female prawns, although this may vary in other seasons. The levels of prevalence and mean infection intensity were low. This study provides essential data on the prevalence, mean infection intensity, and occurrence of these parasite species in *M*. *amazonicum*.

## References

[B001] Barros MSF, Silva LS, Calado TCS (2021). First record of parasitism by *Probopyrus pandalicola* (Isopoda, Bopyridae) on the freshwater prawn *Macrobrachium acanthurus* (Decapoda, Palaemonidae) and ecological interactions. J Parasit Dis.

[B002] Beck JT (1980). Larval and adult habitats of a branchial bopyrid *Probopyrus pandalicola* on one of its freshwater shrimp hosts *Palaemonetes paludosus.*. Crustaceana.

[B003] Beck JT (1979). Population interactions between a parasitic castrator, *Probopyrus pandalicola* (Isopoda: Bopyridae), and one of its freshwater shrimp hosts, *Palaemonetes paludosus* (Decapoda: Caridea). Parasitology.

[B004] Boxshall G, Lester R, Grygier M, Høeg J, Glenner H, Shields J, Rohde K (2005). Marine Parasitology..

[B005] Boyko CB, Williams JD, Martin JW, Crandall KA, Felder DL (2009). Decapod Crustacean Phylogenetics.

[B006] Brasil-Lima IM, Young P (1998). Catalogue of Crustacea of Brazil..

[B007] Bush AO, Lafferty KD, Lotz JM, Shostak AW (1997). Parasitology meets ecology on its own terms: Margolis et al. revisited. J Parasitol.

[B008] Calado R, Bartilotti C, Goy JW, Dinis MT (2008). Parasitic castration of the stenopodid shrimp *Stenopus hispidus* (Decapoda: Stenopodidae) induced by the bopyrid isopod *Argeiopsis inhacae* (Isopoda: Bopyridae). J Mar Biol Assoc U K.

[B009] Carvalho MR, Ebach MC, Williams DM, Nihei SS, Trefaut Rodrigues M, Grant T (2014). Does counting species count as taxonomy? On misrepresenting systematics, yet again. Cladistics.

[B010] Chaplin-Ebanks SA, Curran MC (2007). Prevalence of the bopyrid isopod *Probopyrus pandalicola* in the grass shrimp, *Palaemonetes pugio*, in four tidal creeks on the South Carolina-Georgia coast. J Parasitol.

[B011] Coelho PA, Ramos-Porto M (1984). Camarões de água doce do Brasil: distribuição geográfica. Rev Bras Zool.

[B012] Collart OO (1990). Interactions Entre Le Parasite *Probopyrus bithynis* (Isopoda, Bopyridae) Et L’Un De Ses Hôtes, La Crevette *Macrobrachium amazonicum* (Decapoda, Palaemonidae). Crustaceana.

[B013] Collart OO (1991). Strategie de reproduction de *Macrobrachium amazonicum* en Amazonie Centrale (Decapoda, Caridea, Palaemonidae). Crustaceana.

[B014] Conner SL, Bauer RT (2010). Infection of adult migratory river shrimps, *Macrobrachium ohione*, by the branchial bopyrid isopod *Probopyrus pandalicola.*. Invertebr Biol.

[B015] Corrêa LL, Sousa EMO, Silva LVF, Adriano EA, Oliveira MSB, Tavares-Dias M (2018). Histopathological alterations in gills of Amazonian shrimp *Macrobrachium amazonicum* parasitized by isopod *Probopyrus bithynis* (Bopyridae). Dis Aquat Organ.

[B016] Dale WE, Anderson G (1982). Comparison of morphologies of *Probopyrus bithynis, P. floridensis*, and *P. pandalicola* larvae reared in culture (Isopoda, Epicaridea). J Crustac Biol.

[B017] Dumbauld BR, Chapman JW, Torchin ME, Kuris AM (2011). Is the collapse of mud shrimp (*Upogebia pugettensis*) populations along the Pacific coast of North America caused by outbreaks of a previously unknown bopyrid isopod parasite (*Orthione griffenis*). Estuaries Coasts.

[B018] Fingerman M (1997). Crustacean endocrinology: a retrospective, prospective, and introspective analysis. Physiol Zool.

[B019] García-Guerrero M, de los Santos Romero R, Vega-Villasante F, Cortes-Jacinto E (2015). Conservation and aquaculture of native freshwater prawns: the case of the cauque river prawn *Macrobrachium americanum* (Bate, 1868). Lat Am J Aquat Res.

[B020] Hassan M, Sharoum FM, Abd Wahid ME, Ghaffar MA, Ambak MA, Musa N (2017). Infestation ff *Probopyrus* sp. on *Macrobrachium lanchesteri* From Sungai Chalok and Sungai Nyatoh, Terengganu, Malaysia. J Sustain Sci Manag.

[B021] Holthuis LB (1952). A general revision of the Palaemonidae (Crustacea Decapoda Natantia) of the Américas. II. The Subfamily Pontoninae. Occasional Papers.

[B022] Ismael D, New MB, New MB, Valenti WC (2000). Freshwater prawn culture: the farming of Macrobrachium rosenbergii..

[B023] Jiménez P, Vargas M (1990). *Probopyrus pandalicola* (Isopoda: Bopyridae) infesting *Palaemonetes hiltonii* (Crustacea: Caridea), along the Pacific coast of Costa Rica. Rev Biol Trop.

[B024] Lemos de Castro A, Loyola e Silva J, Schaden R (1985). Manual de identificação de invertebrados límnicos do Brasil.

[B025] Lester RJG, Rohde K (2005). Marine parasitology..

[B026] Maciel CR, Valenti WC (2009). Biology, fisheries, and aquaculture of the Amazon River prawn *Macrobrachium amazonicum*: a review. Nauplius.

[B027] Marin Jarrin JR, Shanks AL (2008). Ecology of a Population of *Lissocrangon Stylirostris* (Caridea: Crangonidae), with Notes on the Occurrence and Biology of its Parasite, *Argeia Pugettensis* (Isopoda: Bopyridae). J Crustac Biol.

[B028] Markham JC (1985). A review of the bopyrid isopods infesting caridean shrimps in the northwestern Atlantic Ocean, with special reference to those collected during the Hourglass Cruises in the Gulf of Mexico. Mem Hourglass Cruises.

[B029] Masunari S, Castagini AS, Oliveira E (2000). The population structure of *Probopyrus floridensis* (Isopoda, Bopyridae), a parasite of *Macrobrachium potiuna* (Decapoda, Palaemonidae) from the Perequê River, Paranaguá basin, southern Brazil. Crustaceana.

[B030] Melo GAS, Magalhães C, Bond-Buckup G, Buckup L (2003). Manual de identificação dos Crustacea Decapoda de água doce do Brasil..

[B031] Muñoz G (1997). Primer registro de isopodos bopyridos (Isopoda: Epicaridea) en el nape *Notiax brachyophthalma* (M. Edwards, 1870) y algunos aspectos de la relación hospedador- parásito. Gayana Oceanol.

[B032] Neves CA, Santos EA, Bainy AC (2000). Reduced superoxide dismutase activity in *Palaemonetes argentinus* (Decapoda, Palemonidae) infected by *Probopyrus ringueleti* (Isopoda, Bopyridae). Dis Aquat Organ.

[B033] New MB, D’Abramo LR, Valenti WC, Singholka S, New MB, Valenti WC (2000). Freshwater prawn culture: the farming of macrobrachium rosenbergii..

[B034] Paul M, Chanda M, Maity J, Gupta S, Patra BC, Dash G (2010). Parasitic prevalences in fresh water prawn *Macrobrachium rosenbergii* in north and South 24 parganas districts of West Bengal. Chron Young Sci.

[B035] Pereira RIS, Maciel CR, Iketani G (2022). Molecular features of *Probopyrus* sp. (Isopoda: Bopyridae) from Brazilian Amazonia and the parasitism of inland populations of *Macrobrachium amazonicum* (Decapoda: Palaemonidae). Parasitology.

[B036] Pralon BGN, Antunes M, Mortari RC, Bueno SLS, Negreiros-Fransozo ML (2018). Infestation of two shrimp species of the genus *Palaemon* Fabricius, 1798 (Decapoda, Palaemonidae) by an isopod of the genus *Probopyrus* Giard & Bonnier, 1888 (Bopyridae) from the Brazilian southeast coast. Nauplius.

[B037] Raman RP, Pagarkar AU, Makesh M (2005). Gupta N. A record of *Probopyrus bithynis* (Richardson, 1904) in *Macrobrachium rosenbergii* (de Man) from coastal Andhra Pradesh, India, with special reference to the host-parasite relationship. J Indian Fish Assoc.

[B038] Rasch JA, Bauer RT (2015). Temporal variation in population structure of the isopod *Urobopyrus processae* Richardson, 1904 (Isopoda: Bopyridae) infesting the branchial chamber of the night shrimp *Ambidexter symmetricus* Manning and Chace, 1971 (Decapoda: Processidae). Nauplius.

[B039] Ribeiro FB, Horch AP, Williams JD (2019). New occurrences and host records for two species of parasitic isopods (Isopoda, Cymothoida, Bopyridae) associated with caridean shrimps (Decapoda, Caridea) from Brazil. J Nat Hist.

[B040] Ribeiro FB, Horch AP (2023). Checklist of parasitic isopods from Brazil: Bopyroidea and Cryptoniscoidea (Isopoda: Cymothoida: Epicaridea). Zootaxa.

[B041] Rocha SS, Bueno SLS (2000). *Probopyrus floridensis* Richardson, 1904 (Isopoda, Bopyridae) parasitizing the freshwater prawn *Macrobrachium potiuna* (Müller, 1880), from São Paulo, Brazil. Nauplius.

[B042] Román-Contreras R, Martínez-Mayén M (2011). Registros nuevos de parásitos epicarideos (Crustacea: Isopoda) en México y suroeste del golfo de México. Rev Mex Biodivers.

[B043] Román-Contreras R, Álvarez F, Rodríguez-Almaraz GA (2008). Crustáceos de México: estado actual de su conocimiento Nuevo León.

[B044] Román-Contreras R (1993). *Probopyrus pacificensis,* a new species of parasite (Isopoda: Bopyridae) of *Macrobrachium tenellum* (Smith, 1871) (Decapoda: Palaemonidae) from the Pacific coast of Mexico. Proc Biol Soc Wash.

[B045] Román-Contreras R, Hendrickx ME (2004). Contributions to the study of east Pacific crustaceans.

[B046] Román-Contreras RA (1996). new species of *Probopyrus* (Isopoda, Bopyridae), parasite of *Macrobrachium americanum* Bate, 1868 (Decapoda, Palaemonidae). Crustaceana.

[B047] Sherman MB, Curran MC (2015). Sexual sterilization of the daggerblade grass shrimp *Palaemonetes pugio* (Decapoda: Palaemonidae) by the bopyrid isopod *Probopyrus pandalicola* (Isopoda: Bopyridae). J Parasitol.

[B048] Shields JD, Boyko CB, Williams JD, Castro P, Davie PJF, Guinot D, Schram FR, von Vaupel Klein JC (2015). Treatise on zoology – anatomy, taxonomy, biology - the Crustacea.

[B049] Subramoniam T (2011). Mechanisms and control of vitellogenesis in crustaceans. Fish Sci.

[B050] Swetha CH, Sainath SB, Reddy PR, Reddy PS (2011). Reproductive endocrinology of female crustaceans: perspective and prospective. J Mar Sci Res Dev.

[B051] Vargas-Ceballos MA, López-Uriarte E, García-Guerrero MU, Vega-Villasante F, Román-Contreras R, Akintola SL (2016). Infestation of *Probopyrus pacificensis* (Isopoda: Bopyridae) in *Macrobrachium tenellum* (Caridea: Palaemonidae) in the Ameca River, Jalisco, Mexico: prevalence and effects on growth. Pan-Am J Aquat Sci.

[B052] Verdi AC (1991). Presencia de *Probopyrus bithynis* Richardson, 1904 em el Uruguai (Isopoda, Epicaridea, Bopyridae). Rev Bras Biol.

[B053] Walker SP (1977). *Probopyrus pandalicola*: discontinuous ingestion of shrimp hemolymph. Exp Parasitol.

[B054] WoRMS (2023). Epi info.

